# Multi-Omics Integration in Stroke: Neuroinflammatory Endotypes, Immune Cell Crosstalk, and Precision Biomarker Discovery

**DOI:** 10.3390/ijms27135984

**Published:** 2026-07-03

**Authors:** Nurittin Ardic, Rasit Dinc

**Affiliations:** 1 Med-International UK Health Agency Ltd., Nuneaton CV11 6LT, UK; nurittinardic@yahoo.com; 2 INVAMED Institute for Medical Innovation, New York, NY 10007, USA

**Keywords:** multi-omics integration, stroke, neuroinflammation, endotypes, microglia, precision medicine, biomarkers, artificial intelligence, blood–brain barrier, immune cell crosstalk

## Abstract

Stroke remains one of the leading causes of death and disability worldwide, yet its clinical management is constrained by substantial biological heterogeneity that single-biomarker and single-omics approaches fail to resolve. The integration of multiple molecular data layers, such as genomics, epigenomics, transcriptomics, proteomics, metabolomics, and immunomics, offers a transformative framework for investigating the underlying neuroinflammatory mechanisms of different stroke subtypes and endotypes. In this review, we synthesize the current multi-omics evidence in stroke by examining how genetic variants propagate through regulatory and immune pathways and generate measurable molecular signatures and clinically relevant biomarkers. We investigate the roles of microglia, infiltrating monocyte-derived macrophages, astrocytes, neutrophils, T cells, and endothelial cells as interacting nodes in the neuroimmune network after stroke, and analyze how spatially resolved single-cell transcriptomics illuminate state-specific programs previously undetectable in bulk tissue analyses. We discuss how proteomics and metabolomics translate these cellular programs into actionable circulating biomarkers and examine emerging evidence on blood–brain barrier disruption and neurovascular unit remodeling as multi-omics-defined targets. We then explore AI and machine learning frameworks enabling the integration of heterogeneous, high-dimensional datasets for endotype classification, patient stratification, and therapeutic response prediction. Finally, we address translational barriers, including analytical standardization, multi-ancestry generalizability, and regulatory readiness, and propose a roadmap for precision stroke medicine based on systems immunology. The core conceptual point of this review is the shift from describing omics findings in stroke cases to redefining biologically meaningful neuroinflammatory endotypes and using multi-omics to enable precision cerebrovascular medicine.

## 1. Introduction

Stroke is the second leading cause of death worldwide and the leading cause of acquired adult disability; contributing to an estimated 12.2 million new cases and 101 million existing cases annually [1]. Despite significant advances in thrombolysis and mechanical thrombectomy, a substantial proportion of patients remain disabled or die, and more than half of those who undergo thrombectomy experience persistent long-term neurological disability [2]. This persistent therapeutic ceiling reflects not a lack of treatment options, but a fundamental challenge in biological heterogeneity: Stroke is not a single disease, but a spectrum of vascular, immunological, and metabolic phenotypes that share overlapping clinical manifestations while differing substantially in their underlying mechanisms, inflammatory trajectories, and therapeutic response [3].

The fundamental paradox of stroke research mirrors the paradox of cardiovascular medicine in a broader sense: Genome-wide association studies (GWAS) have identified dozens of susceptibility loci encompassing vascular integrity, lipid metabolism, inflammation, and atherogenesis, yet translating these genetic signals into clinically applicable biomarkers or therapeutics has been challenging [4,5]. The variant-phenotype gap reflects the layered complexity of stroke pathogenesis, where most risk alleles map to non-coding regulatory regions whose effects are context-specific, cell-type-dependent, and modified by environmental and immune status dynamics [6]. No single analytical method can close this gap. The integration of multi-omics layers, such as genomics, epigenomics, transcriptomics, proteomics, metabolomics, and immunomics, provides a systematic framework for tracking how inherited genetic predisposition propagates through molecular networks and generates measurable, clinically relevant phenotypes [7,8].

Neuroinflammation is now considered a fundamental and active driver of both stroke injury and recovery, rather than a secondary correlation of neuronal damage [9,10]. The post-stroke immune response involves rapid activation of resident microglia cells in the brain, sequential recruitment of circulating neutrophils, monocytes, and lymphocytes, and disruption of the blood–brain barrier (BBB). Bidirectional communication occurs between central and peripheral immune compartments and can persist for weeks or even months after the acute event. Critically, these neuroimmune dynamics are heterogeneous: the magnitude, composition, and temporal evolution of the inflammatory response differ profoundly among patients, stroke subtypes, lesion locations, and comorbidities, influencing trajectories of recovery [11,12]. Understanding this heterogeneity at molecular resolution is a prerequisite for developing stratified therapies that target specific inflammatory pathways at the right time in the right patients.

The emergence of single-cell and spatially resolved transcriptomics has transformed studies of neuroinflammatory biology by enabling the direct characterization of cell-type-specific gene programs in intact tissue architecture [13]. Longitudinal single-cell atlases of brain and blood after stroke reveal the dynamically evolving transcriptional states of different microglia, macrophages, neutrophils, and endothelial cells along the ischemia–reperfusion timeline, providing a biological basis for endotype classification previously unattainable by aggregate analyses [12,14]. In parallel, large-scale plasma proteomics and metabolomics platforms identify circulating signatures that capture systemic immune-metabolic dysregulation and offer potential translational utility as precision biomarkers [15,16]. Artificial intelligence and machine learning are becoming increasingly important for synthesizing these heterogeneous, high-dimensional datasets and extracting reproducible predictive signals applicable to clinical decision support [17,18].

Given the complexity and biological heterogeneity of stroke-induced neuroinflammation, the fundamental conceptual challenge is not merely the accumulation of additional omics datasets, but the integration of these molecular layers into mechanistically interpretable disease frameworks. The core premise guiding this review is summarized in Box 1.

Box 1 **Core conceptual premise of the review.**

  The Core Premise of This Review: Rather than describing omics findings related to stroke, this review asks: How can multi-omics integration redefine biologically meaningful neuroinflammatory stroke endotypes and enable precision cerebrovascular medicine? The answer requires a progression from single-layer biomarker discovery to systems immunology frameworks that link genetic predisposition, cellular immune programs, molecular intermediates, and clinical phenotypes in an integrated, mechanistically interpretable architecture.

**Figure 1:**
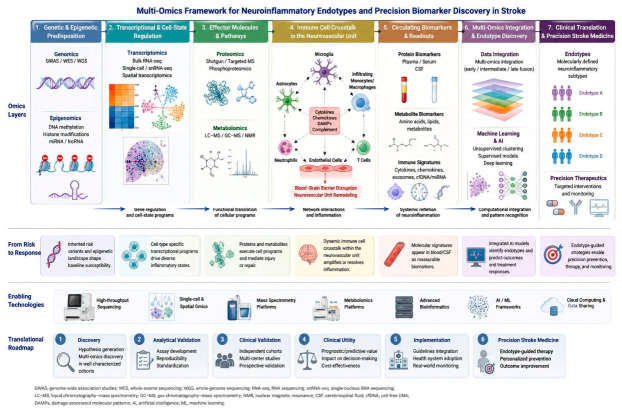
Multi-omics framework for neuroinflammatory endotype discovery and precision biomarker discovery in stroke. An integrated overview of the multi-omics architecture underlying stroke neuroinflammatory heterogeneity and precision biomarker discovery. The framework illustrates how inherited genetic and epigenetic predisposition propagates through transcriptomic cell state regulation, proteomic and metabolomic effector pathways, neuroimmune cell crosstalk within the neurovascular unit, and circulating biomarker production, enabling AI-assisted endotype discovery and precision stroke medicine. The figure highlights the sequential transition from molecular predisposition to clinically applicable neuroinflammatory endotypes by integrating genomics, epigenomics, transcriptomics, proteomics, metabolomics, immunomics, and computational modeling. Translational implementation pathways, including analytical validation, clinical validation, and precision therapeutic stratification, are also shown. The figure was originally created by the authors. Blue arrows indicate information flow across omics layers; colored panels represent individual omics domains. Abbreviation: miRNA, microRNA.

In this review, we synthesize current evidence regarding the integration of multi-omics in stroke treatment using a precision medicine approach. We begin by examining the clinical heterogeneity of stroke and the rationale for neuroinflammatory endotyping (Section 2). We then investigate each major omics layer and its contribution to stroke pathobiology (Section 3). By integrating single-cell and spatial evidence, we analyze immune cell crosstalk along the neuroimmune axis (Section 4). We explore biomarker and endotype discovery from integrated multi-omics datasets (Section 5). We examine artificial intelligence and machine learning frameworks for multi-omics integration and clinical translation (Section 6). Finally, we address key translation barriers currently limiting clinical adoption (Section 7) and propose a forward-looking precision medicine roadmap (Section 8). To provide a conceptual overview of the system-level framework addressed in this review, Figure 1 summarizes how genetic predisposition, immune cell state transitions, molecular effectors, circulating biomarkers, and AI-assisted multi-omics integration come together to identify biologically meaningful neuroinflammatory endotypes in stroke and support precision cerebrovascular medicine.

## 2. Clinical Heterogeneity of Stroke and the Importance of Neuroinflammatory Endotyping

Ischemic stroke encompasses numerous pathophysiological subtypes that differ substantially in upstream mechanisms, immune activation profiles, and therapeutic sensitivities: large artery atherosclerosis, cardioembolic events, small vessel occlusion, cryptogenic stroke, and stroke of other established etiologies [19]. Hemorrhagic stroke, including intracerebral hemorrhage and subarachnoid hemorrhage, introduces further mechanistic differentiation through mass effect, hemoglobin-mediated toxicity, and differing patterns of glial and immune cell activation. Within each etiological category, marked intra-patient variability exists in inflammatory load, lesion volume, penumbra salvageability, and long-term recovery that current patient stratification systems do not adequately capture.

The concept of neuroinflammatory endotyping arises from the recognition that clinically similar stroke presentations may result from biologically distinct inflammatory programs. This approach is becoming increasingly aligned with precision medicine frameworks in cerebrovascular disease [20]. A patient whose stroke is driven by immunothrombotic neutrophil–platelet interactions may respond differently to anti-inflammatory strategies than a patient with predominantly mismatched microglial activation or impaired efferocytosis. Endotype classification requires biomarkers that capture causal inflammatory pathways rather than non-specific indicators of tissue damage.

Multi-omics integration is particularly well-suited for endotype discovery because different omics layers capture complementary aspects of inflammatory heterogeneity. Genomics identifies inherited predisposition and enables causal inference. Epigenomics reveals regulatory status and environment–gene interactions; transcriptomics captures active gene programs and cellular state transitions; proteomics identifies functional effectors and circulating signatures; metabolomics reflects systemic biochemical outcomes; and immunomics resolves immune cell composition and activation states. Together, these layers can identify patient subgroups with shared multi-omics profiles that define mechanistically consistent endotypes and are amenable to targeted intervention; this framework has begun to bear clinical fruit as large-scale multi-omics stroke cohorts mature [2,8,17].

### Cerebral Small Vessel Disease: A New Multi-Omics Target

While much of the current stroke multi-omics literature focuses on acute ischemic stroke, increasing attention is being directed toward cerebral small vessel disease (CSVD), which is a major contributor to ischemic stroke, vascular cognitive impairment, and dementia. CSVD is increasingly recognized as a complex neurovascular disorder involving endothelial dysfunction, chronic neuroinflammation, BBB disruption, immune signaling abnormalities, and perivascular pathology [21,22]. These mechanisms overlap substantially with the biological pathways discussed throughout this review and support the concept that CSVD represents a chronic neuroinflammatory endotype in the broader spectrum of cerebrovascular disease.

Recent multi-omics studies have begun to characterize molecular signatures associated with endothelial activation, extracellular matrix remodeling, vascular senescence, and inflammatory pathway dysregulation in CSVD. Integrated transcriptomic, proteomic, metabolomic, and neuroimaging analyzes have revealed biological links between CSVD progression, cognitive decline, and neurodegenerative disease pathways [21,23]. These observations suggest that systems-level molecular profiling may improve risk stratification and facilitate the identification of biologically distinct CSVD subgroups.

Future studies integrating multi-omics datasets with advanced neuroimaging biomarkers may enable earlier detection of disease, improve mechanistic classification, and support the development of targeted preventive and treatment strategies for patients with CSVD and related vascular cognitive disorders.

## 3. Multi-Omics Layers in Stroke: From Genetic Architecture to Molecular Phenotypes

### 3.1. Genomics: Heritable Risk Architecture and Stroke Susceptibility Loci

The genetic architecture of stroke is complex and polygenic. Large-scale GWASs have identified more than 40 susceptibility loci, encompassing vascular integrity genes (PITX2, ZFHX3 for atrial fibrillation-associated stroke), lipid metabolism (SORT1, PCSK9 pathway), inflammation (9p21/ANRIL), epigenetic regulation (HDAC9), and small vessel biology (FOXF2, ARID1B) [24,25]. A multi-ancestry meta-analysis integrating over 520,000 participants identified 32 loci associated with stroke and revealed significant heterogeneity in effects across ancestries and sexes, underscoring the need for diverse cohort representation in future exploratory studies [25].

Most GWAS signals localize to non-coding regulatory regions, requiring integration with epigenomic and functional data to identify causal variants and effector genes. Interpreting these loci increasingly requires integrative functional genomics approaches that can link regulatory variants to cell-specific biological programs [26]. A multiome-wide association study integrating GWAS in human brain and vascular tissues with single-nucleus chromatin accessibility and gene expression data identified novel effector genes and tissue-specific regulatory mechanisms underlying stroke risk loci [6]. Polygenic risk scores (PRS) derived from multi-ancestry GWAS data have begun to show clinical utility for early stroke risk stratification. However, generalizability across non-European ancestry and the ability to capture the full spectrum of rare variant contributions remain limitations requiring active methodological development [4,5].

Mendelian randomization (MR) studies have leveraged genetic tools to establish causal relationships between inflammatory mediators, lipid types, and stroke risk, providing a biological basis for biomarker prioritization independent of observational confounding [27]. In the context of stroke, MR has identified specific cytokine pathways, coagulation factors, and lipoprotein subfractions as causally related to different stroke subtypes, offering a genetically grounded framework for multi-omics biomarker development [5].

### 3.2. Epigenomics: Regulatory State, DNA Methylation, and Non-Coding Variant Interpretation

Epigenomic profiling provides the necessary regulatory context to interpret non-coding genetic risk variants and understand how environmental exposures, age, and inflammation reshape gene programs in stroke-related cell types. Epigenome-wide association studies (EWAS) have identified DNA methylation loci associated with stroke risk and outcome; recent analyses highlight those regulatory elements in immune and endothelial cell populations are particularly rich in stroke-associated signals [28]. HDAC9 is among the most consistently replicated stroke susceptibility loci among stroke susceptibility loci, encodes a histone deacetylase whose risk variants affect chromatin accessibility and transcriptional programs in vascular smooth muscle and immune cells; this demonstrates how epigenomic mechanisms translate genetic risk into cellular phenotypes associated with atherosclerosis and plaque instability [5].

In the context of acute stroke, epigenomic state transitions occur rapidly in active microglia, infiltrating myeloid cells, and endothelial cells, reshaping gene regulatory programs that determine the balance between pro-inflammatory damage amplification and pro-resolution repair [9]. Single-cell chromatin accessibility profiling (scATAC-seq) is beginning to map these state transitions at cell-type resolution, revealing how inflammatory stimuli mechanistically reshape regulatory environments between microglia subsets and myeloid cell states [13]. These epigenomic maps form a critical bridge between inherited genetic risk, environmental alterations, and active transcriptional programs driving neuroinflammatory heterogeneity.

### 3.3. Transcriptomics: Gene Expression Programs, Single-Cell Atlases, and Spatial Resolution

Transcriptomics has provided some of the most transformative information regarding stroke neuroinflammation in the last three years, with the widespread adoption of single-cell RNA sequencing (scRNA-seq) and spatially resolved transcriptomics. A groundbreaking study by Garcia-Bonilla et al. [12] created a comprehensive atlas of post-stroke immune dynamics by performing longitudinal single-cell transcriptomic analyses from the brain and blood of mice 2 and 14 days after ischemic stroke. The study revealed that microglia, monocyte-derived macrophages, and neutrophil transcriptomes differentiated strongly over time, while endothelial cells and border-associated macrophages showed distinct signatures 2 days after stroke. Time-trajectory inference predicts in situ transdifferentiation of blood monocytes into brain macrophage phenotypes, whereas neutrophils are continuously recruited de novo. These distinct trajectories may have important therapeutic implications. [12]. Single-cell technologies now enable the simultaneous characterization of transcriptional states, chromatin accessibility, and cellular lineage trajectories at unprecedented resolution [29].

Spatial transcriptomic studies have added a significant tissue architecture dimension to these cellular atlases. Spatial–temporal transcriptomic mapping of glial cell responses in acute brain ischemia in mice has identified microenvironment-specific gene programs in peri-infarct and penumbral regions [30], while parallel studies integrating spatial transcriptomics and aggregate proteomics in post-stroke reperfusion have documented early disruption of blood–brain barrier integrity via claudin-5 downregulation and IL-6 upregulation in peri-infarct endothelial cells [31]. Post-stroke astrocyte responses have been mapped through integrated single-cell and spatial transcriptomics, revealing regional heterogeneity in reactive astrogliosis programs; different reparative and neurotoxic astrocyte states occupy different spatial niches around the infarct nucleus [32].

At the functional level, single-cell transcriptomic data have identified metabolically reprogrammed microglial subsets characterized by active glycolysis, lipid metabolism alterations, and suppression of oxidative phosphorylation after ischemic injury; the transcription factor ATF3 has emerged as a key regulator of ischemia-associated microglial gene programs. Blood transcriptomic studies in human stroke patients reveal peripheral immune signatures reflecting central neuroinflammatory processes via the brain-peripheral immune axis and provide a practical sampling framework for multi-omics biomarker capture in clinical settings [33].

Despite their transformative impact, single-cell and spatial omics technologies are still associated with several significant limitations. Current platforms are characterized by high acquisition costs, limited accessibility, substantial computational requirements, and susceptibility to batch effects and sample processing bias. While tissue dissociation procedures can alter cellular transcriptional states, variations in sequencing depth, spatial resolution, and analytical pipelines can reduce cross-study reproducibility. Furthermore, translating highly granular molecular findings into clinically actionable biomarkers remains challenging, as many defined cellular states lack standardized validation frameworks and prospective clinical evaluation. These limitations must be considered when interpreting emerging single-cell and spatial omics discoveries in stroke research.

### 3.4. Proteomics: Circulating Agents, Post-Translational Modifications, and Biomarker Candidates

Proteomics bridges the gap between intracellular gene programs and clinically accessible circulating signatures, creating a critical translational layer within the multi-omics framework. Large-scale plasma proteomics platforms using data-independent acquisition mass spectrometry and affinity-based methods can now measure thousands of proteins from small-volume clinical samples, enabling systematic mapping of the circulating proteome at multiple time points in stroke patients [15,34]. A major portion of circulating proteins is under partial genetic control, and the localization and MR integration of protein quantitative trait loci (pQTLs) with stroke GWAS data has identified candidate causal proteins that can serve as both biomarkers and therapeutic targets, including complement pathway components, coagulation factors, and immune effectors.

Post-translational modifications (PTMs) are of particular mechanistic importance in the context of stroke, as they capture dynamic inflammatory and thrombotic processes that are not visible in transcriptomic analyses. The citrullination, phosphorylation, and oxidation of inflammatory proteins reflect states of active neutrophil, platelet, and endothelial activation that directly contribute to vascular occlusion and disruption of the blood–brain barrier. Multi-omics studies integrating plasma proteomics with transcriptomic and immunophenotyping data have begun to identify coherent inflammatory modules. These modules include co-organized networks of cytokines, adhesion molecules, and matrix metalloproteinases. Their circulating protein signatures are tracked by neurological outcome measures, enabling endotype-specific biomarker panels [35,36].

### 3.5. Metabolomics: Systemic Biochemical Signatures of Neuroinflammatory Stress

Metabolomics captures the downstream biochemical consequences of genomics, transcriptomics, and proteomic disruptions, thus providing a functional system-level output of disease biology [37]. A systematic review of metabolomics-based biomarkers in ischemic stroke, synthesizing 51 studies, identified consistent associations between altered amino acid profiles (decreased proline, isoleucine, valine, alanine; increased tyrosine, glutamine, phenylalanine), sphingolipid metabolism, and lactate/glucose dysregulation with stroke onset and prognosis [16]. Certain combinations of metabolites, including serine, isoleucine, betaine, and lysophospholipid species, demonstrated high sensitivity in predicting acute ischemic stroke in exploratory cohorts (training AUC 0.988), but external validation in various populations is necessary before clinical application [16].

From a neuroinflammatory perspective, metabolomic profiles can differentiate between stroke subtypes and capture immune-metabolic endotypes reflecting the interaction between peripheral metabolic disease, systemic inflammation, and cerebrovascular damage. Ceramides and sphingolipid species, already recognized as predictors of cardiovascular events [38], show promise as stroke risk biomarkers capturing convergent lipid-inflammation biology. In the context of multi-omics integration, metabolomic data acts as a convergence layer where upstream genetic and transcriptional disruptions emerge as coherent biochemical patterns, strengthening the underlying mechanistic inference of candidate biomarkers [7,39].

### 3.6. Immunomics: High-Dimensional Immune Profiling and Endotype Resolution

Immunomics resolves the cellular and functional composition of the immune response in stroke with a resolution that captures the heterogeneity defining the endotype. High-parameter flow cytometry, mass cytometry (CyTOF), and single-cell multimodal approaches have identified expansions of active classical monocyte subsets, aberrant T cell phenotypes, and different neutrophil activation states as features of acute stroke associated with neurological outcome and systemic infection complications [9,10]. Longitudinal immunophenotyping studies have documented a post-stroke immunosuppression syndrome characterized by lymphocytopenia, monocyte deactivation, and impaired innate immune function, predisposing patients to pneumonia and urinary tract infections, independently worsening prognosis.

The integration of immunomics with transcriptomic and proteomic layers enables the identification of mechanistically coherent immune modules whose signatures can be translated into simplified circulating biomarker panels. For example, neutrophil–lymphocyte ratio and monocyte subpopulation distributions, readily measurable in clinical settings, have been shown to reflect broader immunomic endotypes identified through high-dimensional profiling. This creates a practical bridge between exploratory immunomics and clinical biomarker application [11]. The major omics layers contributing to stroke neuroinflammatory endotyping differ in terms of their biological resolution, analytical output, and translational applications. Table 1 summarizes the principal omics domains, representative technologies, and their contributions to mechanistically informed stroke endotype classification.

Taken together, these omics layers provide complementary biological information that cannot be captured through single-platform analyses alone.

## 4. Neuroimmune Cell Crosstalk: Microglia, Infiltrating Myeloid Cells, Astrocytes, and Neurovascular Unit

**Figure 2:**
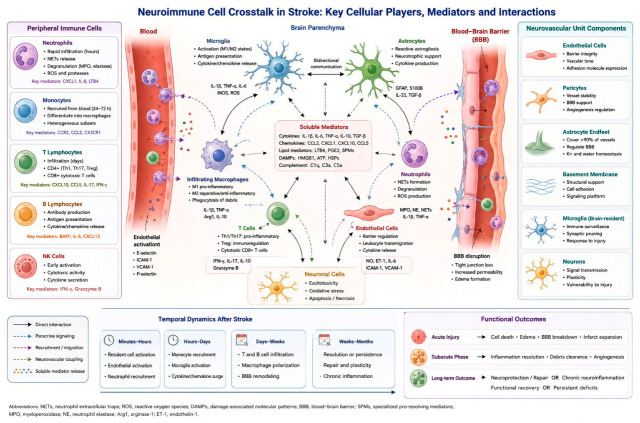
Neuroimmune cell crosstalk after stroke: key cellular interactions and inflammatory pathways. Schematic overview of the neuroimmune network activated after stroke illustrates the interactions between resident central nervous system cells, infiltrating peripheral immune populations, vascular components, and soluble inflammatory mediators. Following disruption of the blood–brain barrier, neutrophils, monocytes, macrophages, T lymphocytes, B lymphocytes, and natural killer cells infiltrate the damaged brain and interact with microglia, astrocytes, endothelial cells, and neurons via cytokines, chemokines, reactive oxygen species, complement factors, and damage-associated molecular patterns. The temporal dynamics of immune activation throughout the acute, subacute, and chronic phases of stroke are shown, along with key functional outcomes including neuroinflammation, blood–brain barrier disruption, tissue repair, and chronic neurodegeneration. The figure highlights the neurovascular unit as a central system-level interface linking peripheral immunity and central nervous system damage. The figure was originally created by the authors. Colored panels distinguish the principal cellular compartments and neurovascular unit components involved in post-stroke neuroimmune interactions, while arrows indicate the direction and type of cellular communication. Abbreviations: BBB, blood–brain barrier; DAMPs, damage-associated molecular patterns; ROS, reactive oxygen species; NETs, neutrophil extracellular traps; SPMs, specialized pro-resolving mediators; NK, natural killer; IL, interleukin; TNF, tumor necrosis factor; IFN, interferon; TGF-β, transforming growth factor beta; VEGF, vascular endothelial growth factor.

Stroke-induced neuroinflammation emerges through dynamic interactions between resident central nervous system cells and peripheral immune populations, linking innate immunity, vascular dysfunction, and tissue damage [40]. Figure 2 summarizes the major cellular players, signaling pathways, and temporal immune dynamics shaping injury and repair after stroke.

### 4.1. Microglia: Sentinel Cells and State-Specific Responses

Microglia are the brain’s resident immune sentinel cells and the first cells to respond to ischemic damage. Single-cell transcriptomic atlases have markedly revised the historical M1/M2 polarization framework, revealing that microglia adopt a range of transcriptional states in the post-stroke brain that do not clearly fit into binary activation categories. At least six microglial subsets have been identified in the post-stroke brain. Among these is a potentially stroke-specific subtype characterized by distinct chemokine, phagocytosis, and metabolic gene programs [14,41]. A single-cell regulatory network analysis identified ATF3 as a key transcription factor regulating ischemia-associated microglial gene expression and linked metabolic reprogramming (active glycolysis, altered lipid metabolism) to neuroinflammatory enhancement and potential opportunities for metabolic immunomodulation.

Temporal dynamics are critical for understanding microglia function after stroke. Two days after stroke, microglia show a strong increase in inflammatory, phagocytic, and danger signal response programs. By day 14, distinct states associated with progressive neurodegeneration and synaptic remodeling emerge [12]. These state-specific programs are not only consequential effects but also active determinants of whether the post-stroke immune environment is geared toward repair or secondary damage. Multi-omics integration has identified microglia-specific molecular modules that can enable blood-based tracking of microglia endotype trajectories in clinical cohorts, including circulating protein surrogates such as TREM2 fragments, soluble TMEM119, and specific chemokine signatures [11].

### 4.2. Infiltrating Myeloid Cells: Monocyte-Derived Macrophages and Neutrophils

Peripheral myeloid cell infiltration begins hours after stroke onset and develops over days to weeks. Monocyte-derived macrophages progressively infiltrate the periinfarct region, where pseudo-time-trajectory analyses predict in situ transdifferentiation into distinct day 2 and day 14 brain macrophage phenotypes with different inflammatory, phagocytic, and reparative programs from blood precursors [12]. This transdifferentiation is shaped by the local microenvironment, including DAMPs, microglia-derived signaling, and neurovascular unit components, which can define pro-resolution and maladaptive macrophage states amenable to therapeutic manipulation.

During the acute stroke period, new neutrophils are continuously recruited from the blood and play a dual role in both exacerbating blood–brain barrier disruption and contributing to early pathogen defense. Cross-species multi-omics analyses of post-stroke brain tissue have identified a myeloid-driven endothelial oxidative stress axis where neutrophil-derived reactive oxygen species enhance endothelial dysfunction and promote secondary infarct expansion. From a multi-omics biomarker perspective, neutrophil-associated plasma proteins, including myeloperoxidase, neutrophil elastase, and matrix metalloproteinase 9, represent accessible circulating effectors of myeloid activation states associated with infarct volume and functional outcome in clinical cohorts [11,42].

### 4.3. Spatial Architecture of Astrocytes, Oligodendrocytes, and Glial Responses

Astrocytes undergo reactive astrogliosis after stroke, shifting to different transcriptional states with differing neurotoxic or neuroprotective functional orientations depending on their spatial location relative to the infarct nucleus. Spatially resolved transcriptomics have revealed that astrocytes in the peri-infarct penumbra adopt gene programs regulating glutamate buffering, water homeostasis, and synaptic plasticity, while astrocytes adjacent to the nucleus express pro-inflammatory and scar-forming programs that can limit both lesion expansion and axonal regeneration [32]. The spatial heterogeneity of astrocyte states creates distinct local signaling environments that shape microglial function, neuronal survival, and vascular repair, which cannot be captured by aggregate or even single-cell analyses lacking spatial context.

### 4.4. Blood–Brain Barrier Disruption: A Target Defined by Multi-Omics

Blood–brain barrier disruption is a central event in stroke pathophysiology, enhancing secondary damage by enabling peripheral immune cell infiltration, albumin leakage, and a disordered neuronal microenvironment. Spatial transcriptomics, combined with bulk proteomics, have identified the early molecular events underlying blood–brain barrier disruption after ischemia–reperfusion and identified claudin-5 (CLDN5) downregulation, actin cytoskeletal degradation, and IL-6-driven pericyte inflammatory activation as convergent mechanisms in peri-infarct endothelial cells [28]. Single-cell transcriptomic profiling of the cerebrovascular region has revealed profound regional heterogeneity in blood–brain barrier (BBB) gene programs among arterial, capillary, and venous endothelial subtypes. Reactive venous endothelial cells, characterized by high adhesion molecule expression and preferential leukocyte transmigration, are key mediators of peripheral immune cell entry [43].

From a multi-omics biomarker perspective, the neurovascular unit represents a particularly valuable multilayered target: its disruption is captured at the transcriptomic level by endothelial state transitions, at the proteomic level by circulating tight junction proteins and matrix metalloproteinases, and at the metabolic level by sphingolipid changes reflecting changes in endothelial membrane composition. The integration of these layers can provide a mechanistically grounded, multidimensional index of blood–brain barrier integrity, and the clinical translational potential of this index is being evaluated in prospective stroke cohorts, including the PROMISE study (NCT05815836) [44].

### 4.5. Glymphatic Dysfunction and Perivascular Clearance Pathways

In addition to BBB disruption, growing evidence indicates that dysfunction of the glymphatic system plays a significant role in stroke-associated neuroinflammation and secondary brain injury [21,22]. The glymphatic pathway is a perivascular fluid transport network that facilitates the exchange of cerebrospinal fluid and interstitial fluid, supporting the clearance of metabolic waste products, inflammatory mediators, and cellular debris from the central nervous system. Disruption of this system following a stroke can exacerbate inflammatory responses, worsen cerebral edema, and impair tissue recovery.

Recent studies have demonstrated links between glymphatic dysfunction, enlarged perivascular spaces, impaired clearance of inflammatory mediators, and post-stroke cognitive decline. MRI-based biomarkers, such as the diffusion tensor imaging along the perivascular space (DTI-ALPS) index, have emerged as promising non-invasive indicators of glymphatic impairment in cerebrovascular disease [45,46]. As glymphatic function is closely linked to astrocyte biology, neurovascular unit integrity, blood–brain barrier status, and immune signaling, it represents a biologically significant target for multi-omics research.

The future integration of spatial transcriptomics, proteomics, metabolomics, advanced neuroimaging, and single-cell analyses could help define glymphatic-related neuroinflammatory endotypes and identify novel therapeutic targets for precision stroke medicine.

## 5. Biomarker Discovery and Endotype Classification from Integrated Multi-Omics Data

### 5.1. Circulating Biomarkers: From Discovery to Patient Stratification

The clinical translation of multi-omics biomarker discovery in stroke requires moving beyond individual marker correlations toward validated multi-marker panels that capture complementary biological axes (inflammation, coagulation, vascular integrity, and metabolic stress) within a coherent endotype framework. A systematic review of emerging biomarkers in ischemic stroke identified numerous candidate classes, including glial fibrillary acidic protein (GFAP), serum S100B, neurofilament light chain (NfL), and a range of inflammatory cytokines and metabolites. However, it noted that few of these have made it into clinical practice due to standardization challenges, pre-analytical variation, and increasing value relative to established risk factors [47,48].

Multi-omics integration approaches have begun to overcome these challenges by identifying signals converging across multiple molecular layers, providing more robust and mechanistically interpretable biomarker candidates than single-layer approaches. Cross-species multi-omics analysis integrating plasma proteomics, single-cell transcriptomics, and functional validation identified a myeloid-derived endothelial oxidative stress signature in ischemic stroke, and this signature converged to specific plasma protein signatures detectable in human samples. A multilayer integrative study combining methylomics, transcriptomics (mRNA, circRNA, miRNA), and graph neural network analysis demonstrated that multi-omics integration improved the accuracy of stroke etiology classification compared to single omics approaches and has potential implications for subtype-specific treatment selection [8].

Recent multi-omics studies integrating transcriptomic, proteomic, metabolomic, and AI-enabled analytical approaches have begun to identify biologically coherent inflammatory signatures and clinically relevant biomarker candidates across different stroke subtypes. Representative studies demonstrating the translational evolution of this field are summarized in Table 2.

All these studies demonstrate that integrating multi-layered molecular data into clinically interpretable stroke endotypes is becoming increasingly feasible.

### 5.2. Endotype Classification: From Research Clusters to Patient Stratification

Identifying biologically meaningful stroke endotypes through multi-omics integration requires unsupervised clustering and network-based approaches that can identify molecular modules co-organized across data layers without imposing predetermined clinical categories. Multi-omics Factor Analysis (MOFA) and related latent variable decomposition approaches can identify sources of variation shared across omics layers corresponding to interpretable biological programs (e.g., inflammatory activation, metabolic stress, or coagulation disorder) and assign patients to endotype clusters based on their multi-layered molecular profiles [7].

One of the most important challenges in translating discovery-level endotypes into clinical use is reducing high-dimensional multi-omics clusters to clinically deployable stratification tools. This transition reflects broader precision medicine frameworks that emphasize biologically informed patient stratification rather than syndromic classification alone [49]. The most promising approaches involve identifying convergent interlayer signals where the transcriptomic activation of an inflammatory program is reflected by corresponding proteomic and metabolomic signatures. The next step is identifying a minimal set of clinically measurable markers (blood proteins, metabolite ratios, or immune cell phenotypes) that adequately capture the endotype signature. Endotypes identified in this way not only improve prognostic accuracy but can also potentially serve as patient selection criteria for clinical trials of anti-inflammatory therapies, addressing a long-standing translational challenge in stroke immunology [9,11].

## 6. Artificial Intelligence and Machine Learning for Multi-Omics Data Integration in Stroke Research

**Figure 3:**
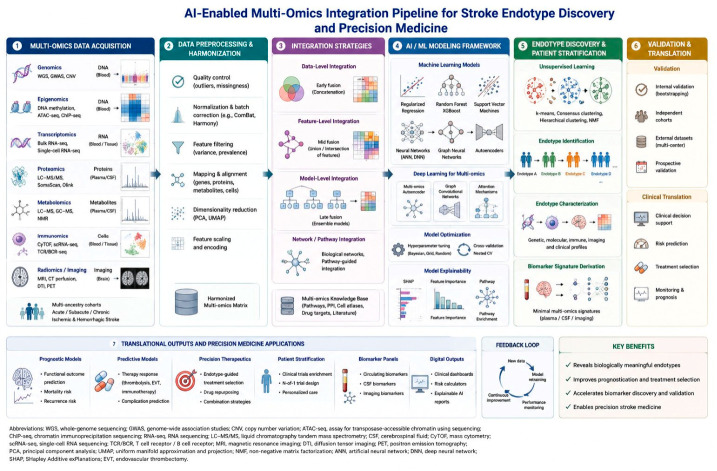
AI-enabled multi-omics integration pipeline for stroke endotype discovery and precision medicine. Overview of computational workflows used for multi-omics integration in stroke research and precision cerebrovascular medicine. Multi-omics layers, including genomics, epigenomics, transcriptomics, proteomics, metabolomics, immunomics, and imaging-derived radiomics, are harmonized through quality control, feature normalization, dimensionality reduction, and batch correction prior to multimodal integration. The figure illustrates early, mid, and late fusion integration strategies, along with representative machine learning and deep learning architectures including random forests, graph neural networks, autoencoders, transformer models, and Bayesian frameworks. AI-powered clustering and latent representation learning enable the identification of biologically meaningful neuroinflammatory endotypes, biomarker signatures, and patient stratification models. Validation pathways, explainability frameworks, and translational applications including clinical decision support systems, prognostic modeling, and precision therapeutic selection are also shown. The figure was originally created by the authors. Colored panels represent sequential stages of the multi-omics integration pipeline, while arrows indicate the analytical workflow from data acquisition to clinical translation. Abbreviations: AI, artificial intelligence; ML, machine learning; CT, computed tomography; PPI, protein–protein interaction; GWAS, genome-wide association study; WGS, whole-genome sequencing; WES, whole-exome sequencing; PCA, principal component analysis; UMAP, uniform manifold approximation and projection; SHAP, Shapley additive explanations; CNN, convolutional neural network; GNN, graph neural network.

Integration of heterogeneous multi-omics datasets into clinically interpretable stroke endotypes requires structured computational workflows encompassing data harmonization, multimodal integration, machine learning modeling, validation, and translational implementation. The convergence of AI and high-dimensional biological data is increasingly viewed as a framework providing a fundamental opportunity for precision medicine and computational disease modeling [50]. Figure 3 summarizes the major AI-powered analytical pipelines currently driving precision stroke multi-omics research.

### 6.1. Integration Strategies and Model Architectures

Stroke multi-omics datasets are characterized by high dimensionality, modality-specific noise structures, and frequent gaps between individuals. These features necessitate AI and machine learning approaches that go beyond traditional biostatistics. A systematic review of machine learning models for stroke risk stratification using multi-omics data identified seven eligible studies (*n* = 40,274) published between 2022 and 2025 that reported combined omics layers through mid-level integration strategies. These strategies most commonly utilized binary groups such as metabolomic–proteomic and metabolomic–lipidomic. The study data showed that supervised machine learning algorithms demonstrated good to excellent accuracy (reported AUCs across studies ranged from 0.78 to 0.99) [17]. This review also identified significant heterogeneity in validation practices and reporting quality. Only four studies included external validation, a critical deficiency given the known risk of overfitting in high-dimensional omics modeling.

Integration strategies generally fall into three categories: early fusion (combining all omics features into a single matrix before modeling), mid-level fusion (learning latent cross-modal representations through matrix factorization, variational autoencoders, or multiple view learning), and late fusion (combining predictions from modality-specific models). Each presents different trade-offs between cross-modal interaction modeling and practical applicability. An integrative multi-omics approach using random forest and artificial neural network models for early stroke diagnosis and immune infiltration characterization achieved high classification performance in internal validation. Random forest models identified multi-omics immune gene signatures associated with stroke outcome [18]. Multidimensional multi-omics approaches targeting ischemic stroke and related chronic pain have extended this framework to post-stroke complications, identifying immune system-related cell death pathways as convergent therapeutic targets [51].

### 6.2. Explainability, Validation, and Regulatory Considerations

The clinical application of AI-powered multi-omics models requires demonstration of analytical validity, clinical validity, and clinical utility in accordance with a hierarchical evidence framework. For AI-powered software as a medical device (SaMD), regulatory expectations from both the FDA and emerging international frameworks emphasize transparent documentation of intended use, representativeness of training data, explainability of model outputs, and continuous post-implementation performance monitoring [52]. Explainability methods, including SHAP-based feature attribution, attention mechanism visualization in LIME and transformer architectures, provide tools for communicating model logic to clinicians and regulators. However, in highly correlated omics domains, these methods require careful interpretation as surrogate features may be reported instead of causal factors.

Reporting standards such as TRIPOD (for multivariate predictive models) and PROBAST (for bias risk assessment) provide a foundation for transparent and reproducible reporting, particularly in multi-omics AI research where risks such as feature leakage between training and validation sets, inappropriate aggregate effect management, and overfitting to study-specific confounding factors are common [53,54]. External validation in multicenter and multi-ancestry cohorts should be treated as a translational requirement, not an optional follow-up, as the tendency for high internal AUC values to fail in independent real-world settings is well-documented. Because stroke multi-omics datasets are highly dimensional and biologically heterogeneous, multiple computational architectures have emerged to support cross-modal integration, endotype discovery, and predictive modeling. The principal AI and machine learning approaches currently applied in stroke multi-omics research are summarized in Table 3.

Although these computational frameworks differ substantially in terms of complexity and interpretability, they collectively demonstrate the expanding methodological toolkit available for precision stroke multi-omics integration.

Despite the encouraging results reported in exploratory studies, significant challenges remain regarding the routine clinical implementation of AI-driven multi-omics models for stroke treatment. Common limitations include dataset bias, underrepresentation of populations with diverse ancestries, limited external validation, model overfitting, and reduced interpretability associated with highly complex deep learning architectures [55]. Many published models have been developed using relatively small cohorts characterized by significant feature-to-sample imbalances, thereby increasing the risk of overly optimistic performance estimates and poor generalizability. Overcoming these challenges will require multi-center collaborative datasets, prospective validation studies, transparent reporting standards, and the continued development of explainable AI methodologies capable of fostering clinician trust and regulatory acceptance.

## 7. Translation Barriers: Standardization, Generalizability, and Clinical Translation

### 7.1. Analytical Standardization and Pre-Analytical Variation

One of the key challenges in translating multi-omics stroke biomarker discoveries into clinical practice is the variability introduced by pre-analytical factors such as sampling time relative to stroke onset, blood processing protocols, freeze–thaw cycles, and platform heterogeneity. These factors can meaningfully alter metabolomic and proteomic profiles independently of the biological signal. Metabolomic biomarkers are particularly sensitive to fasting status, sample processing temperature, and anticoagulant type, requiring rigorous protocol standardization for multicenter studies [16]. Proteomic platforms differ in terms of dynamic range, antibody specificity, and sensitivity to matrix effects, and systematic comparisons of aptamer-based and mass spectrometry-based proteomics across population cohorts have demonstrated both complementarity and major discrepancies that need to be considered in inter-study integration [15].

### 7.2. Multi-Ancestry Representation and Generalizability

The current multi-omics stroke literature heavily weights populations of European ancestry, creating a fundamental generalizability challenge. Polygenic stroke risk scores derived from GWAS show markedly impaired predictive performance in non-European ancestry due to linkage disequilibrium structures, allele frequencies, and differences in local genetic architecture [4,5]. Improving ancestral diversity in genomic and multi-omics studies is therefore essential for robust external validation, preventing population-specific algorithmic bias, and ensuring the equitable implementation of precision cerebrovascular medicine [56]. Metabolomic and proteomic profiles, which are predictive in European cohorts, may perform differently in populations with different dietary habits, comorbidity histories, and genetic origins. Addressing this gap requires the deliberate diversification of stroke multi-omics cohorts, the harmonization of data collection protocols in ethnically diverse settings, and the development of ancestry-sensitive analytical approaches that account for population-level confounding factors [57].

### 7.3. Clinical Scalability and Application Pathways

The high dimensionality (typically encompassing hundreds to thousands of features) of exploratory-level multi-omics panels presents challenges for clinical applications where test complexity, processing time, and cost must be compatible with acute stroke care workflows. Translating multi-omics endotype signatures into viable clinical tools often requires reduction to minimal marker panels that maintain mechanistic specificity: a few blood proteins, metabolite ratios, or simplified immune phenotype indices that can be rapidly measured from standard clinical samples. The pathway from multi-omics exploration to clinical application requires sequential demonstration of analytical validity (reproducibility, sensitivity, reference ranges), clinical validity (association with meaningful outcomes in relevant populations), and clinical utility (improved patient management and outcomes in prospective studies) [47,48].

### 7.4. Health-Economic and Implementation Considerations

Beyond scientific validation, the successful implementation of multi-omics precision medicine in stroke treatment will require demonstrating economic feasibility and health system readiness. Comprehensive multi-omics profiling remains a costly approach, necessitating advanced laboratory infrastructure, specialized bioinformatics expertise, and significant computational resources [58]. These requirements may limit accessibility in resource-constrained health systems and contribute to disparities in the implementation of precision medicine.

The clinical adoption of multi-omics biomarkers will also depend on reimbursement frameworks, cost-effectiveness analyses, integration into existing clinical workflows, and the demonstration of added value beyond established diagnostic and prognostic tools [59]. Prospective studies evaluating real-world clinical utility, patient outcomes, and economic impact will be essential before widespread implementation can be justified. The development of scalable and cost-effective biomarker panels derived from discovery-level multi-omics datasets could represent a crucial intermediate step toward routine clinical practice.

In addition to analytical and regulatory challenges, practical implementation barriers could significantly impact the clinical adoption of multi-omics stroke biomarkers. Many advanced technologies remain costly and require specialized laboratory infrastructure, bioinformatics expertise, and high-performance computing resources. These technologies include methodological approaches such as single-cell sequencing, spatial transcriptomics, and large-scale proteomic profiling [60]. Consequently, the use of comprehensive multi-omics platforms may be limited in resource-constrained healthcare systems. Therefore, future translational efforts should incorporate health-economic evaluations alongside analytical validation to determine whether biomarker-driven approaches yield sufficient clinical benefit relative to their implementation costs. Multicenter prospective studies should also assess scalability across healthcare settings with varying levels of technological capacity. Developing simplified biomarker panels and cost-effective analytical workflows may be essential to ensure equitable access to precision stroke medicine across diverse populations and healthcare environments.

## 8. Precision Medicine Roadmap for Multi-Omics Integration in Stroke Treatment

The combination of single-cell multi-omics, spatial transcriptomics, large-scale proteomics, AI-powered data integration, and genetically based causal inference creates an unprecedented opportunity to redefine stroke as a collection of biologically distinct neuroinflammatory endotypes rather than a single disease requiring undifferentiated treatment. Realizing this opportunity requires progress across six linked domains.

1. Harmonized multi-omics stroke cohorts: Large-scale, prospectively assembled biobanks with standardized multi-omics sampling at defined time points across acute, subacute, and chronic phases are essential for robust endotype discovery and cross-population validation.
2. Genetically anchored biomarker prioritization: Candidate biomarkers should be prioritized using convergent genetic evidence, including pQTL colocalization, MR, and GWAS-epigenome integration, to distinguish causal mediators from reactive downstream signatures.
3. Single-cell and spatial multi-omics reference atlases: Human post-stroke brain and blood atlases from clinically well-characterized samples, supported by matched genetic and clinical outcome data, will help translate mouse model discoveries into human endotype biology.
4. AI-enabled endotype classification tools: ML models should follow TRIPOD and PROBAST guidelines, with external validation in multicenter, multi-ancestry cohorts, predefined explainability reporting, and post-implementation performance monitoring aligned with SaMD regulatory expectations.
5. Biomarker-stratified clinical trial design: Clinical trials should enrich for specific endotype populations using validated biomarker entry criteria, rather than enrolling unselected stroke populations in whom treatment effects may be diluted by biological heterogeneity.
6. Precision neuroimmunology as an intellectual framework: The field should shift from viewing inflammation as a complication of stroke to viewing neuroinflammatory endotypes as determinants of stroke biology.

**Figure 4:**
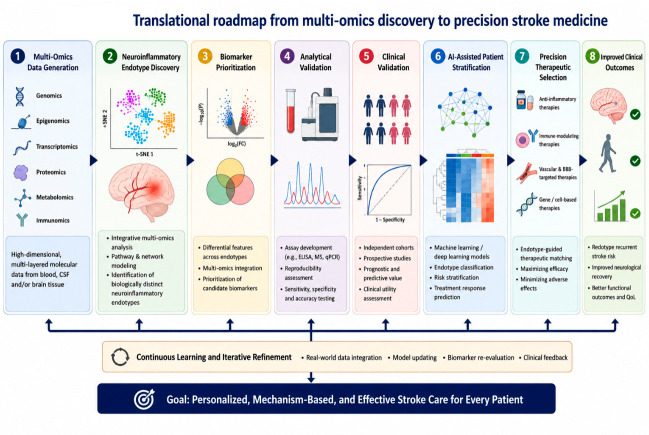
Translational roadmap from multi-omics discovery to precision stroke medicine. A conceptual framework illustrating the progression from multi-omics data acquisition and neuroinflammatory endotype identification to biomarker prioritization, analytical and clinical validation, AI-driven patient classification, precision treatment selection, and improved clinical outcomes. The roadmap highlights the key translational steps required to translate discovery-level molecular insights into clinically actionable precision medicine strategies for stroke. Colored panels represent consecutive stages of the translational roadmap from multi-omics data generation to clinical implementation, while arrows indicate the sequential workflow and continuous feedback between stages. Abbreviations: AI, artificial intelligence; ECG, electrocardiography; MRI, magnetic resonance imaging; CT, computed tomography; ELISA, enzyme-linked immunosorbent assay; MS, mass spectrometry.

To summarize the translational process addressed throughout this review, Figure 4 illustrates the progression from multi-omics data generation and the discovery of neuroinflammatory endotypes in stroke care to biomarker validation, AI-assisted patient classification, and the implementation of precision therapy.

## 9. Conclusions

Multi-omics integration offers the most powerful current framework for resolving the biological heterogeneity defining stroke cases, enabling the identification of neuroinflammatory endotypes, the discovery of mechanistically based biomarkers, and the development of AI-powered precision tools for patient classification and therapeutic targeting. The rapid advancements in the fields of single-cell transcriptomics, spatial omics, proteomics, and metabolomics have transformed precision cerebrovascular medicine from a conceptual goal into a realistic, near-term objective. ML now provides the necessary computational framework to integrate these high-dimensional datasets into clinically interpretable models.

The advancements examined here form the basis of a systems immunology approach to stroke, including longitudinal single-cell immune atlases, spatial transcriptomic mapping of neurovascular unit disruption, and AI-powered multi-omics integration frameworks. At the heart of this transition lies not the accumulation of additional omics findings, but the systematic use of multi-omics to redefine what stroke is biologically: a collection of neuroinflammatory endotypes that are a prerequisite for precision cerebrovascular medicine.

Achieving this vision requires sustained investment in harmonized multi-ancestry cohorts, genetically anchored biomarker prioritization, rigorous AI model validation, and biomarker stratified clinical trial design. The field has reached a turning point where the necessary tools now exist. What remains is the collective commitment to apply them in a coordinated, equitable, and translationally rigorous manner.

## Figures and Tables

**Table 1:** Multi-omics data layers and their roles in stroke neuroinflammatory endotyping.

Omic Layer	Key Technologies	Primary Output	Role in Stroke Endotyping	References
Genomics	WGS, GWAS, SNP arrays, PRS	Genetic variants, susceptibility loci	Inherited risk stratification, causal inference, variant annotation	[4,5,21,22]
Epigenomics	ATAC-seq, ChIP-seq, EWAS	Chromatin accessibility, DNA methylation	Regulatory interpretation of non-coding variants, immune state transitions	[25,26]
Transcriptomics	Bulk RNA-seq, scRNA-seq, spatial transcriptomics	Gene expression programs, cell states	Single-cell endotype mapping, temporal immune dynamics, cell crosstalk	[12,13,14,27,28,29,30]
Proteomics	DIA-MS, LC-MS/MS, affinity-based platforms	Protein abundance, PTMs	Circulating biomarker discovery, functional effector identification, pQTL integration	[15,28,31,33]
Metabolomics/Lipidomics	LC-MS, NMR	Metabolite and lipid profiles	Systemic biochemical endotypes, lipid-inflammation convergence	[16,34,35,36]
Immunomics	CyTOF, flow cytometry, scRNA-seq multimodal	Immune cell composition and activation states	Inflammatory endotype resolution, therapeutic target identification	[9,10,37,39]

Abbreviations: GWAS, genome-wide association study; PRS, polygenic risk score; scRNA-seq, single-cell RNA sequencing; PTMs, post-translational modifications; CyTOF, cytometry by time-of-flight; WGS, whole-genome sequencing; SNP, single nucleotide polymorphism; ATAC-seq, assay for transposase-accessible chromatin using sequencing; ChIP-seq, chromatin immunoprecipitation sequencing; DIA-MS, data-independent acquisition mass spectrometry; LC-MS/MS, liquid chromatography–tandem mass spectrometry; NMR, nuclear magnetic resonance.

**Table 2:** Representative multi-omics studies in stroke neuroinflammation and biomarker discovery (2022–2026).

Cohort/Model	Validation Status	Omics Layer	Key Finding	Translational Output	References
Post-stroke brain and blood atlas	Discovery study	scRNA-seq (brain and blood)	Temporal immune divergence and monocyte-to-macrophage transdifferentiation after stroke	Cellular endotype atlas and therapeutic timing targets	[12]
Integrated multi-omics stroke cohort	Internal validation	Genomics, methylation, mRNA, circRNA, miRNA + GNN	Multi-omics integration improves stroke etiology classification compared to single-layer approach	Multi-marker framework for subtype stratification	[8]
Acute ischemic stroke tissue samples	Discovery study	Spatial transcriptomics	Spatial-temporal mapping of glial activation in acute ischemia	Region-specific neuroinflammatory endotype signatures	[27]
Reperfusion stroke cohort	Independent validation	Spatial transcriptomics + proteomics	CLDN5 downregulation and IL-6-associated BBB disruption after reperfusion	Multi-omics index of BBB integrity	[28]
Systematic review	Literature synthesis	Metabolomics systematic review	Amino acid and sphingolipid signatures associated with ischemic stroke subtypes	Metabolite panel for ischemic stroke prediction	[16]
Multi-cohort ML datasets	External validation	Machine learning across metabolomics, proteomics, and lipidomics	Multi-omics ML improves stroke risk stratification accuracy	AI-assisted integration framework for clinical ML models	[17]
Conceptual precision medicine framework	Translational framework	Multi-omics biomarkers + AI	Dynamic multi-omics profiling supports personalized stroke care	AI-assisted precision stroke management pathway	[2]

Abbreviations: AI, artificial intelligence; BBB, blood–brain barrier; circRNA, circular RNA; CLDN5, claudin-5; GNN, graph neural network; IL-6, interleukin-6; miRNA, microRNA; ML, machine learning; mRNA, messenger RNA; scRNA-seq, single-cell RNA sequencing.

**Table 3:** Representative AI and machine learning approaches for multi-omics integration in stroke research.

Method	Integration Strategy	Strengths	Limitations	Application in Stroke	References
LASSO/Elastic Net	Early fusion	Interpretable, low risk of overfitting	Linear, misses interactions	Biomarker panel selection, risk score construction	[17,20]
Random Forest/XGBoost	Early/late fusion	Nonlinear, handles noise	Limited interpretability, requires tuning	Stroke outcome prediction, immune infiltration profiling	[17,18]
Multi-view learning (MOFA)	Mid-level fusion	Preserves modality structure, cross-layer correlation	Computationally intensive	Endotype discovery, cross-omics disease subtyping	[8,13]
Variational Autoencoders	Mid-level fusion	Latent shared structure, handles missing data	Reduced biological interpretability	Cross-omics dimensionality reduction	[17,45]
Graph Neural Networks (GNN)	Mid-level fusion	Incorporates biological networks	Dependent on network quality	Stroke etiology classification	[17,46]
Transformer/Attention Models	Early/mid fusion	Long-range dependencies modeling, multimodal integration	Requires large training datasets	Dynamic multi-omics biomarker trajectories	[17,45]
Bayesian Networks	Mid/late fusion	Quantitative uncertainty estimation, causal reasoning	Computational complexity	Probabilistic endotype classification	[18,47]

## Data Availability

No new data were created or analyzed in this study. Data sharing is not applicable to this article.
